# The Influence of Dietary Factors on the Gut Microbiota

**DOI:** 10.3390/microorganisms10071368

**Published:** 2022-07-07

**Authors:** Esther Nova, Sonia Gómez-Martinez, Rocio González-Soltero

**Affiliations:** 1 Institute of Food Science and Technology and Nutrition (ICTAN), CSIC, 28040 Madrid, Spain; sgomez@ictan.csic.es; 2 Department of Medicine, Facultad de Ciencias Biomédicas y Salud, Universidad Europea de Madrid, 28670 Villaviciosa de Odón, Spain; mariadelrocio.gonzalez@universidadeuropea.es

**Keywords:** dietary patterns, gut microbiota, macronutrients, culinary methods, chrononutrition

## Abstract

There is increasing evidence that diet influences the relationship between gut microbiota and individual health outcomes. Nutrient intake affects the composition of the gut microbial community and provides metabolites that influence the host physiology. Dietary patterns, including macronutrient balance and feeding/fasting cycles which may be manipulated with dietary regimens based on caloric restriction periods, influence the gut homeostasis through its impact on the microbial ecosystem. Along the same line, prebiotic and probiotic ingredients and additives in foods, as well as the degree of food processing have consequences on gut microbiota and the related immune and metabolic response of the human host. Acquiring knowledge of these aspects, especially through an -omics-integral approach, might provide the basis for personalized nutritional interventions directed to avoid dysbiosis and contribute to the prevention of major chronic degenerative diseases. Despite vast scientific evidence supporting the relationship between dietary factors and gut microbiota composition and function, the underlying mechanisms and their potential impact are far from clear. There is a lack of well-designed longitudinal studies performed in target population groups whose dietary patterns can be particularly relevant for their future health, as is the case in infants, pregnant women, or athletes.

## 1. Introduction

It is now accepted that all eukaryotes are meta-organisms and must be considered together with their microbiota as an inseparable functional unit [1]. The different microbial communities along the gastrointestinal tract directly utilize nutrients and water for their maintenance, and extract energy through the metabolic use of remnant protein and carbohydrates after host digestion. Thus, diet is a fundamental contributor in shaping the gut microbiota. The aim of this perspective article is, then, to provide a non-comprehensive overview of all dietary factors that potentially modulate the stability, shifts, composition, and function of the gut microbiota, which can include dietary regimes, macronutrient profile, food and cooking choices, food additives, and diets for specific population groups and special circumstances, such as feeding alternatives for infants and children, pregnancy, sports nutrition, etc.

To this end, a literature search was performed with key word combinations including gut microbiota (permanent term), diet, macronutrients, chronobiology, food processing, food cooking, food additives, weaning, infant feeding, pregnancy, sports, and exercise in the PubMed database. Some cites were retrieved by using more specific terms and even specific authors of known relevance in particular topics.

The maintenance of a stable gut microbiome ecosystem depends not only on nutrient availability for growth, but also on the microorganism population density, communicated through the microbiota quorum sensing systems [2]. In addition, host factors, including digestive enzymes, peristalsis, and excretion, all contribute to cyclical changes in gut microbiota density and diversity during the day [3]. These allow their dynamic equilibrium as growth and loss of bacterial mass. It is believed that the microorganisms may, in some way, manipulate host mechanisms of energy intake and align with feeding behaviors and interoceptive hunger and satiety. Indeed, several bacterial components and metabolites are detected in the systemic circulation, which may act directly on hypothalamic neurons [4].

Eating rhythms and adherence to a particular dietary pattern exert important effects on the availability of microbial-activity-derived ligands of dietary and non-dietary nature in the luminal content, affecting gut-to-brain sensing routes and controlling food intake and energy metabolism [5]. The gut-brain-axis, involving the microbiota, is believed to affect the control of hypothalamic neuron activity and to be associated with eating behavior and eating disorders, specifically those related to energy balance, such as obesity, anorexia nervosa, and infant malnutrition [6]. These are the interrelationships between diet, behavior, and the microbiota which work in all specific conditions of the human being. The symbiotic interaction between gut microbiota and the host is determinant for human health. Thus, many inflammatory diseases, as is the case of inflammatory bowel disease (IBD), have been associated with dysbiosis [7]. In patients with IBD, gut dysbiosis is accompanied by disruption of diet–microbe interactions that may influence the efficacy of dietary intervention. Intestinal inflammation changes the microenvironment in the gut, including nutrient availability and oxygen levels, which, in turn, alters the composition and function of the bacterial community in the gut [8].

The complexity of the human diet, and the great influence that social and cultural factors have on it, results in the lack of conclusive studies on the suitability of the diet to maintain a healthy microbiota [9]. On the other hand, further studies are needed beyond the composition of the microbiota, relating the components of the diet to the functionality of the microbial community [10]. In this line, the development of Next Generation Sequencing (NGS) methods has allowed the study of microbial communities in greater depth, and as a result, the taxonomic structure of the intestinal microbiome can be analyzed on a routine basis, but methods to infer the metabolic potential of the community are lacking. New applications in the area, including glycomics, proteomics, or the development of new bioinformatics tools such as the CAZy (Carbohydrate-Active enZymes) database, focused on the catalytic activity promoted by carbohydrates, are aimed to fill this gap of knowledge about gut microbiota functionality [11]. This knowledge will allow us to obtain a more precise vision of the integration of the microbiome with the patients’ physiology [12].

A recent review from Klassen et al. [13] pointed out how visualizing microbiome–carbohydrate interactions is possible with carbohydrate probes and could enable rapid identification of carbohydrate interactions at the single cell level.

The complex interactions between phenotype and genotype must also be taken into account when interpreting the composition of the microbiome and its relationship to diet. With this focus, Bailén et al. [14] suggest an association between sugar metabolism and the PPARGC1A rs 8192678 (G/A) genotype and support the notion of specific microbiota signatures as factors related to the onset of Type 2 diabetes mellitus.

## 2. Dietary Regimes, Chronobiology, and the Gut Microbiota

The body and each individual cell undergo circadian rhythms which are interconnected with the daily feeding/fasting cycles. The microbiota is immersed in these waves both as a trigger of signals and as a response element. In addition, due to the close relationship between the microbiota and the immune system, the immune system is also involved in synchronizing circadian clocks; both convey information derived from external cues. The gut microbiome orchestrates host diurnal rhythms in the gut and other digestive glands and organs by incorporating various signals from nutrients, hormones, and innate immune sensing [15].

A master clock located in the mammalian central nervous system (CNS), and the peripheral clocks in every tissue and organ control the circadian rhythms [16]. Thus, chrononutrition is an approach to coordinating the daily rhythms of the body with the timing of food intake [15]. Different dietary regimes are being studied for their capacity to regulate metabolism through several pathways that involve an influence on the daily cycles of neuroendocrine molecules and intermediate metabolites, which ultimately results in synchronization or desynchronization of circadian clocks and shifting of biological processes underlying the development of chronic pathological conditions [15,16,17,18].

**Figure 1:**
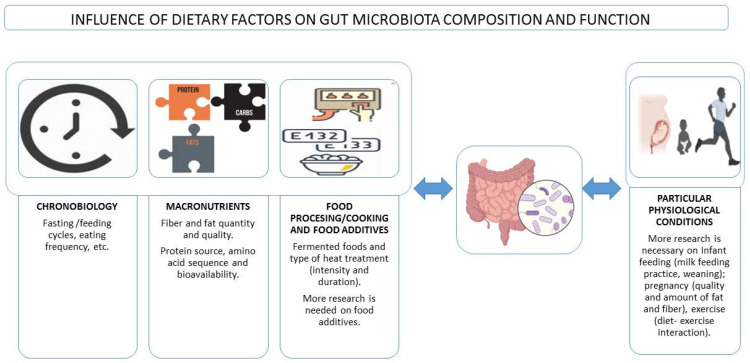
Influence of dietary factors on gut microbiota composition and function and interactions with particular physiological conditions.

In animals and in humans, the composition and function of the gut microbiota vary throughout the day, and this is associated with eating behaviors, including eating frequency and the overnight-fast duration [17] (Figure 1). The microbiota is actively involved in the interplay between the dietary timing and the circadian rhythms by mechanisms involving, among others, the expression of circadian clock genes and epigenetic modifications that can act locally and at distant sites such as the liver [18]. Moreover, it is not only the gut microbiota that shows this shifting pattern, also the immune system seems to change in response to diurnal dietary regimes or the timing of food intake, with subsequent effects on the defense mechanisms against infections, and even impaired immunity towards specific microorganisms. However, these effects are still controversial [19]. In this sense, it is well known that a nutritional regulation exists over the redistribution of immune cells in the organs during fasting and feeding states, and it is speculated that this trafficking might be linked to microbial signals, although how the microbiota impacts the neuronal–immune networks in the gut remains largely unexplored [15].

## 3. Macronutrients–Microbiota Interactions

Digestible carbohydrates are broken down in the small intestine and rapidly released as glucose into the bloodstream. The non-digestible carbohydrates, known as dietary fiber, reach the large intestine. Fermentable fibers, known as microbiota-accessible carbohydrates (MACs), are easily fermented by bacteria in the colon and result in the production of short-chain fatty acids (SCFA), which include acetate, propionate, and butyrate [20]. Fiber-mediated nutrition of gut microbiota also protects against diet-induced obesity by restoring IL-22-mediated colon health [21]. Indeed, pronounced changes in bacterial diversity and the production of microbiota-derived fecal fermentation end products have been demonstrated in as little as 24 h in humans who switched from a diet rich in fiber (>30 g/day) to a meat-based diet devoid of fiber [22]. A diet deprived of fiber can be determinant for breaking the homeostasis associated with health and initiating pathways towards disease [23]. Improving the gut microbiota profile by personalized nutrition and supplementation known to improve immunity can be one of the prophylactic ways by which the impact of immunosenescence can be minimized in older people and to enhance immune response in immuno-compromised patients. Among the strategies more studied are the inclusion of prebiotics/probiotics in the diet, along with current therapies, as it has been suggested even in the case of COVID-19 patients and other viral respiratory infections [24,25]. Safety, however, is a priority and attention should be paid to each patient’s specific condition; for instance, probiotics should not be used in patients with sepsis.

Changes in the diet by increasing the non-digestible fiber inulin, was found to decrease the *Bilophila* genus abundance and to be associated with both softer stools and a favorable change in constipation-specific quality-of-life measures [26]. However, increased fiber may not be adequate in some digestive disorders such as irritable bowel syndrome (IBS). In this group of patients, the integration of a low-FODMAP (fermentable oligosaccharides, disaccharides, monosaccharides, and polyols) diet with probiotics seems to counteract gut microbiota imbalances and, in particular, restore *Bifidobacterium* levels [27].

Dietary fat quantity and quality also influence gut microbiota composition [28] (Figure 1). In mice, a high-fat and low-fiber diet consistent with a Western diet pattern results in a decrease in *Bacteroidetes* and an increase in *Firmicutes* and *Proteobacteria* [29]. Similar results were found in a study by Bailén et al., establishing that the microbiota of, a priori, healthy people who follow a low-fiber, high-fat diet, presents patterns that could be considered as early predictors of inflammatory diseases [23]. In humans, a high intake of dietary fat is associated with lower diversity and richness [30] and correlates with cardiometabolic risk factors following a 6-months dietary intervention [31], especially when the diet includes mainly saturated fatty acids. A highly saturated fat-rich diet was, indeed, associated to some bacterial taxa (*Anaerotruncus, Lachnospiraceae, Flavonifractor, Campylobacter, Erysipelotrichaceae,* and *Eisenbergiella*) that could be related to the development of some diseases, especially obesity and other pro-inflammatory diseases in women [23]. It seems that high-fat diets in healthy adults increase the levels of *Alistipes* and *Bacteroides* species and decrease the presence of *Faecalibacterium*. However, a very low-carbohydrate and high-fat diet, known as ketogenic diet (VLCKD) has been used as a dietary protocol in the last century to treat refractory epilepsy and is gaining potential to treat obesity and other metabolic disorders [32]. However, little is known about its impact on the gut microbiome. *Akkermansia* and *Parabacteroides* are increased in mice following a ketogenic diet [32], and it seems to improve the neurovascular state [33].

On the contrary, the consumption of omega-3 PUFAs leads to an increase in butyrate-producing bacteria and anti-inflammatory effects [34], an increase of *Bifidobacterium* and *Lactobacillus* species, and concomitant SCFA production [35]. Vegans and vegetarians’ microbiota is characterized by a higher alpha diversity compared to omnivores and some operational taxonomic units (OTUs) known to be protective, such as *Bacteoridetes*, *Prevotella,* and *Roseburia,* seem to be increased. A diet based on whole-plant food consumption appears to up-regulate beneficial commensal bacteria and may contribute towards the health effects [36]. The Mediterranean Diet (MD) consists mainly of vegetables, unrefined cereals, fruits, legumes, fish, and olive oil, is combined with moderate consumption of eggs, poultry, dairy products, and a low consumption of refined sugars and red meat. The MD is associated with a reduction in total cardiovascular events, and in the incidence of cancer, Alzheimer’s, and Parkinson’s diseases. Several foods included in the MD show direct anti-inflammatory effects, e.g., the use of extra virgin olive oil is associated with a reduction in thromboxane 2 and leukotriene B4 [37]. The MD also includes a small daily consumption of red wine that contains polyphenols. Polyphenols are bioactive food components that interact with the gut microbiota, modifying the bioavailability of the fiber, which is the main source of energy for colonic fermentation. Polyphenol-rich chocolate and certain fruits have also been shown to increase fecal Bifidobacteria [38]. In addition, a study of 147 healthy adults has shown that a class of polyphenols, which are non-extractable with aqueous organic solvents, and thus known as non-extractable polyphenols (NEPP), have a significant relationship with the *Bacteroides–Prevotella–Porphyromonas* group and *Bifdobacterium* levels in feces [39]. This seems to be related to their enhanced bioavailability by the action of the gut microbiota.

Concerning proteins, the amino acid composition, the source, and its availability to be digested are important clues in determining the microbiota composition. In response to the alterations in dietary proteins, there are also significant changes in the microbial metabolites including SCFAs [40]. A long-term animal-protein-rich diet is associated with the abundance of *Alistipes*, *Bilophila,* and *Bacteroides* and with decreased levels of *Roseburia*, *Eubacterium rectale,* and *Ruminococcus bromii* [22].When the source of protein is mainly plants, Lactobacilli and Bifidobacteria are more abundant and also the observed SCFA production [41].

Probiotics, as represented by Lactobacilli and Bifidobacteria, are considered a dietary factor able to modulate the gut microbiome, host immune system, and gut–brain connection [42]. The benefit of fermented milk in human diet has been acknowledged for centuries, and the bacteria in yogurt and fermented milk products constitute the most important source of probiotics for humans [43]. Ingestion of probiotics such as cheese, yogurt or fermented milk, has been shown to have a health promoting effect on the oral tissues, including a reduction of the incidence of dental caries and the improved management of periodontitis and oral infections [44]. Probiotic supplementation also confers several health benefits by the restoration of the gut microbiota as it has been proposed in the case of, for example, autism spectrum disorders [45] or some others neurological or psychiatric disorders [46].

The widespread consumption of alcoholic beverages among the global population is another dietary factor with potential effects on the gut ecosystem. Scientific evidence is strong under abusive consumption. Specifically, a maintained high consumption of alcohol can lead to dysbiosis associated with bacterial overgrowth, a decrease in *Bacteroidetes,* and an increase in *Proteobacteria* and the families *Enterococacceae* and *Streptococacceae*, among other changes, and these are associated with inflammatory processes [47,48]. The effect of alcoholic beverages on the health is the result of the direct damage from ethanol plus the involvement of gut dysbiosis [49]. For instance, fecal microbiota transplantation from patients with alcoholic hepatitis to humanized germ-free mice induced severe liver inflammation and necrosis by alcohol intake in the animals [50]. Moreover, gut fermentation in the auto-brewery syndrome, leading to high blood ethanol, without alcohol intake, has revealed the involvement of gut bacteria and mycobiota in the spontaneous development of non-alcoholic fatty liver disease [49]. This supports that targeting the gut microbiota is promising for treating alcohol-related liver disease [48,49]. However, moderate consumption of fermented beverages containing microbes, bioactive compounds, and low amounts of alcohol may have other, potentially healthy effects on microbial growth and metabolite production [49,51].

Future research into the diet–gut microbiota-generated metabolites should be considered in the future, taking into account their important role in the link between microbiome profile and human health. Nowadays, we know that changes in diet rapidly alters the human gut microbiota [22]. However, change in diet must be significant, as mild changes in nutritional components seem to not easily disrupt the internal coherence of gut microbiota communities. There is also a personal component in the changes induced by diet as many other factors as age, sex, ethnicity, or medication contribute to shape the gut microbiota composition [52].

## 4. Food Processing/Cooking and Gut Microbiota

Food processing is aimed to produce food products that are both safe and organoleptically acceptable. Treatments can be microbiological, physical, and chemical and are used to increase the shelf-life of the food and/or improve its taste, to improve its appearance, and sometimes even to optimize its nutritional value. Fermentation is an example of microbiological processing widely used in the food industry that is accompanied by a structural change in the food, and frequently, also in the nutritional profile [53]. It is currently accepted that fermented foods can influence the gut microbiota, since on the one hand, the final product may contain microorganisms with a probiotic effect, and on the other hand, certain bioactive peptides are produced during fermentation and some anti-nutrients are reduced [54] (Figure 1). Heat treatments and/or cooking cause major changes in the structure and composition of the food. In fact, heat alters the physicochemical properties of foods and can degrade antimicrobial compounds naturally present in foods [55]. Because of this treatment, new compounds are produced, mostly derived from the Maillard reaction, including melanoidins, which, due to their possible prebiotic role, affect the composition of the gut microbiota. For example, in animals fed melanoidin-enriched malts, a relative decrease in bacterial groups such as *Dorea*, *Oscillibacter,* and *Alistipes* and an increase in *Lactobacillus*, *Parasutterella*, *Akkermansia*, *Bifidobacterium,* and *Barnesiella* have been observed [56].

Several studies have shown that the type of processing and heat intensity used with foods play a role in the observed microbiome changes after their fermentation. Shen et al. [57] used an in vitro faecal batch culture with healthy donors’ microbiota as a model of gut fermentation. Feeding the system with different types of meat products, such as chicken, beef, and fish (salmon), the authors concluded that both the type of meat and the cooking process could influence the fermentation profiles within the human gut microbiota. Recently, Perez-Burillo et al. [58] evaluated the effect of different common culinary methods such as frying, boiling, roasting, grilling, and broiling, on different types of foods (chicken, banana, red pepper, bread, and chickpeas) in an in vitro digestion-fermentation system. These authors observed that foods such as bread, raw banana, and boiled chickpeas produced a healthier gut microbial community, characterized by a higher abundance of some beneficial bacteria such as *Roseburia*, *Ruminococcus*, *Bifidobacterium*, *Dialister*, *Collinsella,* or *Barnesiella*.

A recent publication by Yu et al. [59] concluded that the effect of food on gut microbiota depends, both, on dietary styles (Western diet and Eastern diet), including the food additives used in its preparation (spicy, sour, or sweet) and the type of heat treatment applied to the food (roasted, steamed, or fried), its intensity and duration (undercooked, medium-, or well-cooked).

## 5. Food Additives and Gut Microbiota

In relation to chemical treatments, the use of indispensable additives in the food industry is currently generating a great deal of debate regarding their safety [60]. The Food and Drug Administration (FDA) in the United States and the European Food Safety Authority (EFSA) in Europe regulate their use. The classification of additives focuses on their technological function, and they can only be used for that purpose. However, most of them have never been evaluated for possible alterations on the gut microbiota, the gut barrier, and the immune system [61]. Accumulating evidence suggests that some food additives may disrupt gut homeostasis, thus contributing to the promotion of tissue-damaging inflammatory responses [62]. Indeed, Abiega-Franyutti et al. [63], in their review, conclude that despite the paucity of published studies, many additives can modify the gut microbiota environment (Figure 1). Furthermore, these authors stress the importance of taking into account that the consumption of these additives in our society is chronic, which can have a medium- and long-term effect on the health of the individual.

Some additives such as salt (sodium chloride) have been studied in greater depth, perhaps due to, both, the high intake in our society, higher than the WHO recommendations (5 g/day), and its relationship with certain pathologies. Several studies in animal models reviewed in the publication by Rinninella et al. [11] report that high doses of salt are associated with changes in the abundance of certain bacterial groups. This set of results shows a decrease in one or several of the following groups: *Lactobacillus* spp., *Oscillibacter*, *Pseudoflavonifractor*, *Clostridium* XIVa, *Johnsonella,* and *Rothia*; and an increased abundance in one or several of these: *Parasutterella* spp., *Erwinia* genus, *Christensenellaceae, Corynebacteriaceae*, *Lachnospiraceae,* and *Ruminococcus* [11]. Miranda et al. [64] describe a decrease in *Lactobacillus* sp. and butyrate production that are associated with changes in immune homeostasis following high salt intake.

As reviewed by Raoul et al. [65], other additives, such as dietary emulsifiers, including lecithins and fatty acid monoglycerides and diglycerides, could induce the dysbiosis associated with an alteration of the intestinal barrier, promoting chronic inflammation and abnormal immune response.

Along the same line, there are not many studies carried out with non-caloric sweeteners in humans, which are additives widely used in the food industry and increasingly consumed also as table additives. Suez et al. [66], in a long-term study, found a possible positive correlation between the consumption of these sweeteners and some bacterial groups. In short-term administration, saccharin and sucralose are the most studied in humans, due to the fact that in vitro experiments and animal models seemed to indicate a relationship between their use and changes in gut microbiota. However, no significant effects have been observed when doses used were lower or equal to the adequate daily intake (ADI), as indicated by Del Pozo et al. [67]. Nevertheless, these authors conclude that it is necessary to broaden the concept of food safety of sucralose and saccharin by re-evaluating toxicity in relation to the effect on gut microbiota and the possible consequences for health maintenance.

## 6. Dietary Pattern in Particular Physiological Conditions and the Microbiota

### 6.1. Infant Feeding and Microbiota Development

It is currently very well known that the infant’s microbiota is shaped by the mode of delivery, the type of lactation, all sorts of environmental exposure (e.g., antibiotic administration, pets, and siblings within the household, etc.) and solid food introduction, among others. These factors influence the number and abundance of relevant taxa in the gut microbiota composition.

Regarding breastfeeding versus formula-feeding in the first weeks and months of life, scientific evidence shows that formula feeding is associated with development of an earlier adult resemblance on the infants’ gut microbiota, increased pro-inflammatory taxa abundance, increased permeability, and translocation [68,69] (Figure 1). It is also known that these feeding alternatives are associated with differences in immune system development, growth, and even disease risk later in life and the gut microbial ecology seems to have a role in these events. The study of the composition of the gut microbiome of 98 newborn/infants during the first year of life showed that cessation of breast-feeding, rather than the introduction of solid food, drives the maturation of the infant’s gut microbiome [69] and the effects of breastfeeding for more than 4 months on the microbiota last over the first year and probably longer due to the development of a more stable microbiota [70]. The second most influential factor for microbiota development in infants is the introduction of the semi-solid diet and the timing of it. This period seems to be associated with an increase in SCFA in feces [71]. In addition, when weaning occurs after the 4 months of age, an increase in taxa of the *Firmicutes* phylum, such as *Ruminococcaceae* and *Faecalibacterium*, has been observed [70]. The authors considered that the higher *Faecalibacterium* abundance in 1-year infants may be due to higher resilience of this early colonizer involved in lactose metabolism when children have had 4 or more months for its establishment while lactating. Thus, there is a necessity to carry out well designed studies and in bigger size populations to unravel the interplay between breast-feeding and semi-solid food introduction, as well as the consequences of alternative methods of complementary feeding such as baby-led weaning, which encourages the early introduction of an adult-type diet. In addition, the importance of food and environmental microbes in shaping the early infant microbiota is highlighted by evidences showing that microbes from the breast milk and the skin areola seed the gut microbiota [72].

Malnutrition, as a consequence of diets poor in certain micronutrients, is found not only in developing countries but also in disadvantaged populations of developed countries. Studies of the microbiota composition in malnourished infants are scarce. It is known that a normal richness of species protects against pathogen invasion and grants essential functions for host health. Malnutrition is associated frequently with diarrhea, decreased richness and maturity index of the microbiota, decreased abundance of strict anaerobes, and less efficient nutrient utilization [73,74].

### 6.2. Pregnancy

It has become clear that the physiological changes that occur during pregnancy are not unrelated to the changing composition of the microbiome. It is believed that the microbial ecosystem changes support a healthy pregnancy [75]. On the other hand, in animal models, exposing the mothers to obesogenic diets during pregnancy results in gut microbiota composition alterations and leads to increased metabolic disturbances in the offspring [76]. Moreover, it has been hypothesized that dietary manipulation during pregnancy may affect the gut microbiome in a favorable way in the management of gestational diabetes, e.g., resistant starch might work as a prebiotic and improve glycemic control, involving potentially the production of SCFAs [77]. Although studies are underway, in humans, it is difficult to study the impact of diet during pregnancy and lactation on the microbiome-dependent regulation of the offspring development and its metabolic markers; the published studies are so far scarce and have small sample sizes. Preliminary evidence, however, showed that adherence to a MD pattern was associated with higher diversity in pregnant women microbiota [78], and another study showed that in obese pregnant women, consumption of recommended levels of fat and fiber resulted in lower level of *Bacteroidetes,* and higher bacterial richness compared to women that had a high-fat, low-fiber intake [79].

### 6.3. Exercise

To date, the studies on the effects of an active lifestyle on the microbiome composition in humans are limited and, especially scarce, are those focused on the interaction between the dietary pattern and the level of exercise performance [80]. Several studies have shown that endurance training interventions change the microbiota composition [81] and that the differences in the gut microbiota between elite and non-elite athletes are significant (Figure 1). In fact, according to one study performed in 19 female rowing athletes, the taxonomical and functional biomarkers of the microbiota are useful to correctly classify these athletes as elite and non-elite, and dietary factors explain part of the variability [82]. Regarding sports, probiotics have been assessed for their effect on physical performance. However, the evidence on their mechanisms of action and effectiveness needs to be shown more clearly [83]. Some studies have revealed preliminary evidence of the interaction between diet and microbiota in athletes. One observational study of bodybuilders and distance runners showed that the high-protein diet of athletes might have effects both on microbiota diversity and taxa abundance at genus level [84], and an interventional study in elite endurance race walkers showed that the low-carbohydrate, high-fat diet followed by the athletes induced changes in specific bacterial taxa of the oral microbiota [85].

In conclusion, targeting dietary features to modulate the microbiota composition might be a suitable method to correct metabolic disturbances towards healthier aging at all physiological conditions across the life span (Figure 1). More research is needed until these strategies can be implemented with effectiveness.

## Data Availability

Not applicable.
